# Early Rotator Cuff Repair Yields Lower Retear Rates and Superior Functional Outcomes: A Systematic Review and Meta-Analysis

**DOI:** 10.3390/jcm14155552

**Published:** 2025-08-06

**Authors:** Alexander Baur, Wesley Lemons, Omar Protzuk, Jonathan Brett Goodloe

**Affiliations:** 1 Department of Research, Liberty University College of Osteopathic Medicine, 306 Liberty View Ln, Lynchburg, VA 24502, USA; 2 Department of Orthopaedics, Virginia Commonwealth University School of Medicine, 1201 E Marshall St. #4-100, Richmond, VA 23298, USA; 3 Department of Orthopaedics, Virginia Commonwealth University Medical Center, 1300 W Broad St. #113, Richmond, VA 23284, USA

**Keywords:** rotator cuff repair, shoulder arthroscopy, early operative repair, full thickness rotator cuff tear

## Abstract

**Background:** Optimal timing for surgery following acute rotator cuff tears remains unclear. This study examines how the timing of arthroscopic rotator cuff repair (RCR) affects retear rates and functional outcomes. **Methods:** This PROSPERO-registered review (CRD42024528249) followed PRISMA guidelines and included randomized trials, and cohort, studies on adults with imaging-confirmed full-thickness rotator cuff tears. Studies lacking timing data or key outcomes were excluded. Risk of bias was assessed using ROBINS-I. Meta-analysis of retear rates was performed comparing surgical timing. Qualitative analysis was conducted classifying results as early-beneficial, delayed-detrimental, or neutral. **Results:** Our review included 13 studies and 871 patients with an average age of 57.9. Meta-analysis of eight studies comparing retear rates between early and delayed RCR demonstrated a significant benefit associated with early intervention risk ratio 0.60 (95% CI: 0.38–0.96). Functional outcomes also favored early intervention with four studies demonstrating significantly greater postoperative functional improvements in the early intervention group. **Conclusions:** Early arthroscopic RCR decreased the rate of retear and improved functional outcomes. No study found early intervention to be detrimental or delayed intervention to be superior. These findings support consideration of early repair when clinically appropriate. Future studies should determine more finite timing guidelines.

## 1. Introduction

Rotator cuff tears are a prevalent source of shoulder pain and disability worldwide, significantly impacting patients’ functional abilities and quality of life and imposing a substantial burden on healthcare systems [1]. Arthroscopic RCR is commonly used to address these injuries by restoring shoulder function and alleviating symptoms. Despite its widespread use, ongoing discussions surround the timing of intervention, especially when comparing outcomes of early versus delayed repairs in different age groups. Notably, while surgery is widely accepted as beneficial for persistent symptoms and functional impairments, the clinical benefit of RCR over conservative treatments remains uncertain, particularly in the general population. For example, a Cochrane systematic review suggests that surgery “probably provides little or no improvement in pain” compared to conservative care in some cases [2]. These findings underscore the importance of refining patient selection criteria to optimize outcomes and healthcare resource use.

Management of rotator cuff tears is complex and influenced by a range of patient-specific factors, including age, comorbidities (e.g., hyperlipidemia, smoking), and tear characteristics, such as size, fatty infiltration, and atrophy visible on MRI, all of which increase the risk of repair failure [3,4,5]. Conservative approaches like physical therapy and analgesics can be effective for some patients, but those with persistent symptoms and higher functional demands often require surgical repair [6,7]. Determining optimal surgical timing is particularly relevant given the growing incidence of surgical management in younger patients, who are more likely to experience traumatic tears. However, a lack of consensus and conflicting evidence contribute to variability in clinical practices.

The primary objective of this systematic review is to determine how the timing of RCR affects retear rates and functional outcomes. We hypothesize that early intervention will yield better functional recovery and lower retear rates.

## 2. Methods

A systematic search strategy was devised to identify relevant literature concerning the outcomes of arthroscopic RCR procedures, with a particular focus on the timing of surgical intervention following acute rotator cuff tears. The protocol for this review followed PRISMA guidelines and was registered on the International Prospective Register of Systematic Reviews (PROSPERO) under the identifier [CRD42024528249] before initiation.

We included randomized controlled trials (RCTs), prospective and retrospective cohort studies. Eligible studies involved adult human patients diagnosed with acute full-thickness rotator cuff tears confirmed through clinical examination, imaging modalities (e.g., MRI, ultrasound), or arthroscopy. Specifically, we concentrated on evaluating arthroscopic RCR or mini-open techniques that compared outcomes of early (acute) versus delayed surgical repair following rotator cuff injury.

Exclusion criteria comprised systematic reviews/meta-analyses, case reports, case series, editorials, commentaries, and conference abstracts due to potential methodological limitations. Studies focusing solely on pediatric populations or polytrauma patients or patients with concomitant shoulder pathologies were excluded. Studies focusing on massive rotator cuff tears were excluded. Additionally, studies lacking relevant outcome measures or sufficient data for analysis were excluded. Lastly, studies with inadequate reporting of methodology, results, or outcomes were excluded to minimize bias and uncertainty.

Our analysis included patient demographics (age), follow-up period, retear rates, and functional outcomes (e.g., patient-reported outcome measures such as American Shoulder and Elbow Surgeons score).

**Figure 1:**
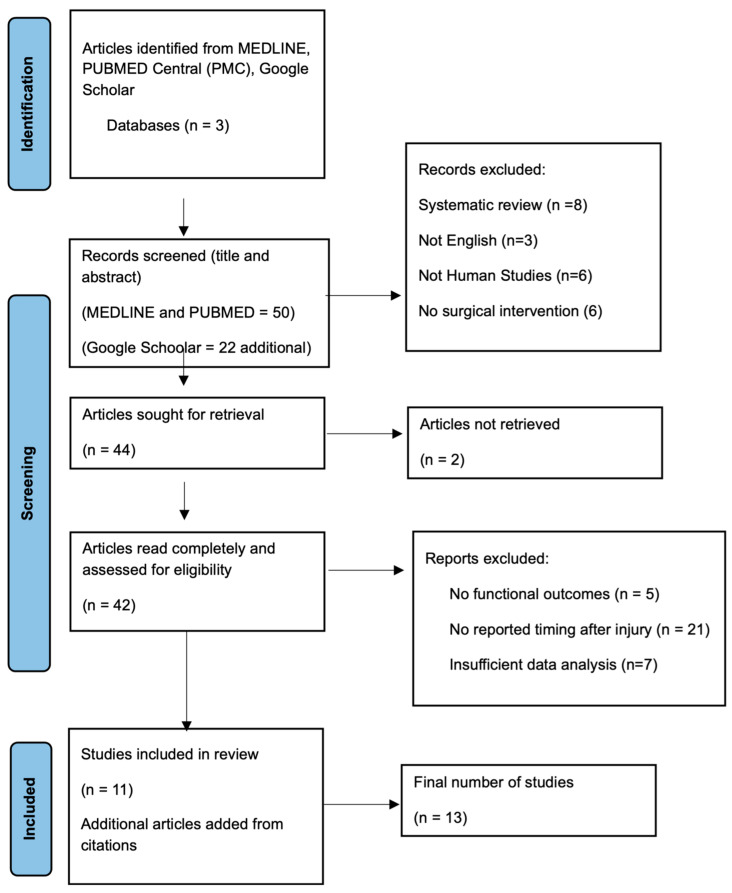
PRISMA Flow Chart.

Initially, a systematic search was conducted across multiple databases, including MEDLINE, PubMed Central (PMC), and Google Scholar. Studies were last searched August 2024. A total of 72 records were identified using the search terms ((“rotator cuff tear” OR “rotator cuff injury”) AND ((“delayed repair” OR “delayed surgery” OR “acute repair” OR “early repair”) OR (“acute injury” OR “chronic injury” OR “nonhealing rotator cuff tear”))). After initial screening of titles, 44 records were retrieved for further evaluation. Following full-text assessment, 13 articles met the eligibility criteria and were included in the systematic review (Figure 1).

Each article in the systematic review was evaluated by two independent reviewers for study design, sample size, age, retear rates, and outcome scores (ASES, Constant, etc.). This information, along with timing conclusions, was meticulously recorded in an Excel spreadsheet (Microsoft Office, version 2024) to ensure organized and consistent data collection. Robins-I risk of bias tool was used to evaluate bias by two separate reviewers and recorded in Table 1. Statistical analysis involved *t*-tests comparing outcome scores and retear rates between younger and older populations. Qualitative analysis included categorizing findings based on pre-specified article conclusions. We conducted a qualitative analysis of the conclusions from the articles to examine clinical significance. The studies that comment on timing were examined and grouped into categories based on predefined parameters of early beneficial, delayed detrimental, or no difference in timing.

## 3. Results

Table 2 summarizes the characteristics of the 871 patients and their reported outcomes. Across all studies, there was a retear rate of 12.5%. The mean age across all studies was 57.9 years old.

**Figure 2:**
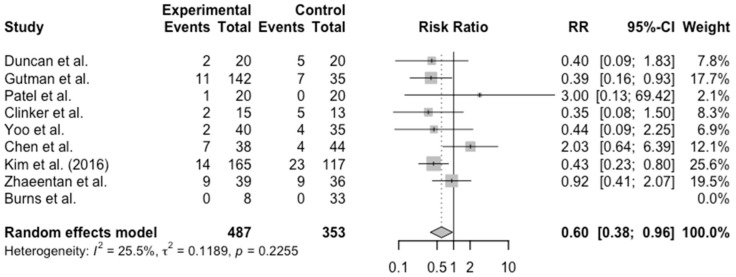
Retear Rate Meta-analysis [8,9,10,12,14,16,17,18,20]. Experimental events: Early intervention. Control Events: Delayed intervention.

Meta-analysis of eight studies comparing retear rates between early and delayed RCR demonstrated a significant benefit associated with early intervention (Figure 2). The pooled risk ratio was 0.60 (95% CI: 0.38–0.96). Heterogeneity was low (I^2^ = 25.5%, *p* = 0.23), suggesting consistency across studies. Overall, the retear rate was 9.9% in the early group and 15.9% in the delayed group. Three studies reported retear rates greater than 20% [8,12,18]. The highest reported rate of retear was 42% in the delayed group of the Clinker et al. study [12].

Four studies identified statistically significant differences between early and delayed intervention groups [9,15,16,17]. The study by Kim et al. found a statistical difference in retear rates between those undergoing surgery less than 1 year after symptoms and those delaying repair [17]. Gutman et al. found that repairs performed within four months were associated with a 10.3-point higher difference in ASES score between preoperative and postoperative (*p* < 0.01) [9]. Hantes et al. observed a 13-point higher difference in preoperative versus postoperative Constant score (*p* < 0.05) for interventions within three weeks compared to delayed intervention [15]. Similarly, Chen et al. reported a nine-point higher postoperative ASES score (*p* < 0.001) in the early intervention group [16].

In analyzing the data, we found many studies to make conclusions supporting early repair even without significant statistical findings. Out of the studies analyzed, six indicated that early intervention was beneficial, three suggested that delayed intervention had negative effects, and four found no significant difference between early and delayed intervention. None of the studies reported early intervention as detrimental, and none found delayed intervention to be advantageous. There was no association with age on timing conclusions (*p* > 0.05).

## 4. Discussion

The primary objective of this systematic review was to assess the impact of early surgical intervention on functional outcomes following arthroscopic RCR. Our results support our hypothesis that early intervention would experience better functional recovery, and lower retear rates. Early intervention consistently achieved higher functional outcomes and lower retear rates. Additionally, our qualitative analysis clearly supported early intervention when possible.

These findings support the clinical practice of early RCR when appropriate. Although not all studies demonstrated statistical significance, the consistent direction of effect and absence of harm associated with early intervention suggest a benefit to timely repair. No study identified early surgery as detrimental, and delayed intervention was never associated with superior outcomes. This consistency across studies provides practical guidance when counseling patients, particularly those with acute tears. Additionally, the lack of association between patient age and study conclusions suggests that the potential benefits of early repair may apply broadly across age groups.

These results highlight the importance of timely recognition and referral for rotator cuff tears. Delays in diagnosis, imaging, or surgical consultation may reduce the likelihood of optimal recovery. Given that early repair was consistently associated with better outcomes and no evidence of harm, clinicians should prioritize early evaluation, particularly for acute or symptomatic tears. This is especially relevant for patients with high functional demands or those hoping to return to work or sport. Establishing efficient care pathways—from initial presentation to surgical scheduling—may improve access to early intervention and help standardize care across institutions.

The limitations of this study are notably influenced by the variability and risk of bias in the included research. Not all studies took into account known prognostic factors for acute RCR outcomes such as tendon fatty infiltration, diabetes, preoperative ROM restriction, and rehab protocol variability. Another important limitation is the variability in how studies define “early” versus “delayed” repair. Timeframes ranged from a few months to 12 months, limiting our ability to draw firm conclusions about optimal timing thresholds. Standardizing these definitions will be crucial in future comparative studies. Additionally, the relatively young average age of patients in the included studies limits the generalizability of these findings. The external validity is limited for older adults, who comprise a substantial proportion of the rotator cuff repair population. Table 1 highlights the heterogeneity across the studies, with many showing high risks of bias due to issues such as lack of matching, poor methodological rigor, and inconsistent statistical analyses. This variability complicates the interpretation of results and underscores the need for cautious conclusions

Moving forward, future studies should explore recovery timelines of early versus delayed interventions, shedding light on whether additional conservative measures expedite post-surgery recovery. Moreover, understanding patient motivation is crucial and is important in the clinical significance discussion. Patients may grow frustrated with conservative measures alone and feel the need for more aggressive intervention. Understanding these underlying dynamics could significantly inform treatment protocols and enhance patient satisfaction and outcomes. Future research endeavors should aim to bridge the gap between clinical efficacy and patient experience, ultimately optimizing orthopedic care delivery.

## 5. Conclusions

Early arthroscopic RCR was associated with lower retear rates and improved functional outcomes in most included studies. Although not all differences were statistically significant, no study found early intervention to be detrimental or delayed intervention to be superior. These findings support consideration of early repair when clinically appropriate and future research into optimizing timing guidelines for RCR.

## Figures and Tables

**Table 1:** Appraisal using the Cochrane ROBINS-I Risk of Bias Assessment Tool.

Author	Confounding	Selection of Participants	Deviations from Intended Interventions	Missing Data	Measurement of Outcomes	Selection of the Reported Result	Overall Bias	Notes
Duncan et al. [8]	+	+	+	+	+	+	Low-Risk	
Gutman et al. [9]	−	+	+	+	+	+	Mod-risk	Lack of matching
Patel et al. [10]	+	+	+	+	+	+	Low-Risk	
Dimmen et al. [11]	−	+	+	+	+	+	Mod-risk	Lack of matching
Clinker et al. [12]	+	+	+	+	+	+	Low-risk	
Sa et al. [13]	−	−	−	+	+	+	High-risk	Lack of matching and variable treatment
Yoo et al. [14]	+	+	+	+	+	+	Low-risk	
Hantes et al. [15]	+	+	+	+	+	+	Low-Risk	
Chen et al. [16]	+	+	?	+	+	+	Uncertain	Cohort study secondary to results of preliminary study
Kim et al. [17]	−	+	+	+	+	+	Mod-risk	Lack of matching
Zhaeentan et al. [18]	+	+	+	+	+	+	Low-risk	
Petersen et al. [19]	−	−	+	−	+	+	High-risk	Lack of matching and robust statistical analyses
Burns et al. [20]	−	+	+	+	+	+	Mod-risk	Lack of matching

Table legend: Low-risk (+); Uncertain (?); High-risk (−). Bolded indicates that the study has a high-risk of bias.

**Table 2:** Study Characteristics.

Study (Author)	Sample Size	Age	Retear Rates	Early/Young Outcomes	Delayed/ Older Outcomes	*p*-Value	Timing Concl
Duncan et al. [8]	40 total <6 mo—20 >18 mo—20	Mean age <6 mo—60 >18 mo—60	2/20 for <6 mo 5/20 for 18 mo	Change in Oxford score Early—20.3	Change in Oxford score Delayed—10.4	*p* = 0.0014	Early beneficial
Gutman et al. [9]	206 total 0–2 mo—66 2–4 mo—76 4–6 mo—29 6–12 mo—35	Mean age 60	0–2 mo—6/66 2–4 mo—5/76 4–6 mo—3/29 6–12 mo—7/35 *p*-value 0.180 (fisher exact)	Change in ASES 0–2 mo—51 2–4 mo—42	Change in ASES 6–12 mo—32	*p* < 0.01	Early beneficial
Patel et al. [10]	40 total <4 mo—20 >4 mo to 2 yr—20	<4 mo—65 >4 mo—65	<4 mo—1/40	Postop Oxford <4 mo—43	Postop Oxford >4 mo—45	*p* > 0.05	Early beneficial
Dimmen et al. [11]	358 total <3 mo—77 >3 mo—281	<3 mo—58 >3 mo—58	NR	Postop WORC <3 mo—43	Postop WORC >3 mo—39	*p* > 0.05	No difference in outcomes based on timing
Clinker et al. [12]	30 total <6 weeks—15 >6 weeks—13	<6 weeks—55 >6 weeks—55	<6 wks—15% >6 wks—42% Pts loss to follow-up	Difference in ASES <6 wks—51	Difference in ASES >6 wks—42	*p* = 0.07	Delayed detrimental
Sa et al. [13]	49 total <6 mo—15 >6 mo—34	<6 mo—56 >6 mo—62		Oxford improvement <6 mo—23	Oxford improvement >6 mo—21	*p* = 0.50	No difference in outcomes based on timing
Yoo et al. [14]	75 total “early”—40 “delayed”—35	“early”—61.5 “delayed”—63.5	“early”—2/40 “delayed”—4/35	Constant score “early”—83	Constant scores “delayed”—88	*p* > 0.05	No difference in outcomes based on timing
Hantes et al. [15]	35 total <3 wks—15 >3 wks—20	<3 wks—54 >3 wks—56		Constant score difference <3 wks—43	Constant score difference >3 wks—30	*p* < 0.05	Early beneficial
Chen et al. [16]	82 total <6 mo—38 >6 mo—44	<6 mo—57 >6 mo—58	<6 mo—7/38 >6 mo—4/44 *p* value = 0.22	ASES <6 mo—91	ASES >6 mo—82	*p* < 0.001	Delayed detrimental
Kim et al. [17]	282 total	<65 y/o—230 >65 y/o—52	>12 mo—19.7% <12 mo—8.5% *p*-value < 0.01				Delayed detrimental
Zhaeentan et al. [18]	75 total <3 mo—39 >3 mo—36	<3 mo—59 >3 mo—59	18/75	Constant scores <3 mo—68	Constant scores >3 mo—69	*p* > 0.05	No difference in outcomes based on timing
Petersen et al. [19]	36 total 0–8 wks—15 9–16 wks—15 >16 wks—6	Avg age 57	NR	ASES 0–8 wks—82 9–16 wks—79 UCLA 0–8 wks—30 9–16 wks—30	ASES >16 wks—65 UCLA >16 wks—25	No stats reported	Early beneficial
Burns et al. [20]	Total 41 (avg time to surgery 13 mo) <3 mo—8 >3 mo—33	Avg age—43.7	0/41	UCLA Postop 32.6 Surgery < 3 mo—32.5	UCLA Postop Surgery > 3 mo—32.9	*p* > 0.05	Early beneficial

## Data Availability

Please contact the corresponding author for any data not included in the manuscript.
